# The applicability of the Beck Depression Inventory and Hamilton Depression Scale in the automatic recognition of depression based on speech signal processing

**DOI:** 10.3389/fpsyt.2022.879896

**Published:** 2022-08-04

**Authors:** Bálint Hajduska-Dér, Gábor Kiss, Dávid Sztahó, Klára Vicsi, Lajos Simon

**Affiliations:** ^1^Department of Psychiatry and Psychotherapy, Semmelweis University, Budapest, Hungary; ^2^Department of Telecommunications and Media Informatics, Budapest University of Technology and Economics, Budapest, Hungary

**Keywords:** depression, machine learning, diagnosis, speech, Support Vector Regression

## Abstract

Depression is a growing problem worldwide, impacting on an increasing number of patients, and also affecting health systems and the global economy. The most common diagnostical rating scales of depression are self-reported or clinician-administered, which differ in the symptoms that they are sampling. Speech is a promising biomarker in the diagnostical assessment of depression, due to non-invasiveness and cost and time efficiency. In our study, we try to achieve a more accurate, sensitive model for determining depression based on speech processing. Regression and classification models were also developed using a machine learning method. During the research, we had access to a large speech database that includes speech samples from depressed and healthy subjects. The database contains the Beck Depression Inventory (BDI) score of each subject and the Hamilton Rating Scale for Depression (HAMD) score of 20% of the subjects. This fact provided an opportunity to compare the usefulness of BDI and HAMD for training models of automatic recognition of depression based on speech signal processing. We found that the estimated values of the acoustic model trained on BDI scores are closer to HAMD assessment than to the BDI scores, and the partial application of HAMD scores instead of BDI scores in training improves the accuracy of automatic recognition of depression.

## Introduction

The growing prevalence of depression is a severe problem in our society, which does not only affect the individual, but it is also burdening health systems and the global economy. WHO studies show that 264 million people are affected by depression today (1). Projections show that depression will be the first cause of burden of disease worldwide by 2030 (2).

Depression is often difficult to diagnose due to the variety of symptoms and the stigma on mental illnesses (3). The most common assessment methods in depression are the Hamilton Rating Scale for Depression (HAMD) and the Beck Depression Inventory (BDI). While the HAMD is a clinician-administered assessment, the evaluation by this instrument can vary depending on the expertise of the clinician, BDI is a self-reported rating scale and patients can exaggerate or conceal symptoms (4).

In the past few decades, several studies attempted to identify biological markers, which could improve the diagnosis and classification of psychiatric disorders (5). Altered levels of serum growth factors and pro-inflammatory cytokines, changes in endocrine factors and metabolic markers can be associated with depression but limited by a lack of sensitivity and specificity (6). Non-invasive EEG biomarkers are also under examination, such as gamma-band power and signal complexity (7).

Speech is a promising biomarker for automatic recognition of depression (8–10). The advantage of speech signal processing is that it is non-invasive and time and cost efficient. Several studies have shown that it is possible to automatically detect depression (11–15) and estimate the severity of depression (9, 16–20) based on speech signal processing.

To support the automatic recognition of depression, it is possible to separate depressed and non-depressed subjects by classification, or to estimate the severity of the subject’s depression using a regression method. Based on literature, it is possible to distinguish between healthy subjects and depressed patients with an accuracy of 60–95%. However, it is difficult to compare the results in the literature in terms of both classification and regression, because the results obtained are influenced by the number of subjects in the examined database (unfortunately, the examined database is generally not public) and the distribution of their severity according to depression. In addition, the possible differences in the scale used to describe the severity of depression (HAMD, BDI, Patient Health Questionnaire 9 – PHQ-9, Montgomery–Åsberg Depression Rating Scale – MADRS) in each experiment further complicate the comparison of regression results. The currently known depressed speech databases are small: the median of the size of the databases (depressed and healthy together) is 123 (10).

Low et al. (10) comprehensive study highlights that in the majority of current studies (62%), self-reported questionnaires were used to determine the severity of depression of examined subjects. The use of self-reported questionnaires is completely understandable, as they allow more data to be obtained more quickly, and a large database is a prerequisite for creating precise machine learning models. Clinician-administered scales, like HAMD and MADRS can assess different symptoms of depression, MADRS can be more sensitive to change in symptoms, but both need more time and professional personnel (21). With the use of self-reported rating scales, the exaggeration and concealment of symptoms as a noise is introduced into the target feature, which degrades the performance of the trained model. The problem of incorrect targets is further exacerbated by the fact that during model optimization, the evaluation of individual models will also be incorrect, which can result in low efficient models, with poor generalizability, due to small size of databases.

The present study examined the extent to which rating scale (HAMD) improves the accuracy of depression estimation. Previous studies show that the correlation between pre-treatment BDI and HAMD scores in depression is between *r* = 0.4 and *r* = 0.7 (22). Several other studies show an increase in the correlation of the rating scales in longitudinal studies, the correlations between observer-rated and self-report scales are 0.4 at the beginning and 0.7 at the end of the study (23). The modest association at the first assessment can be related to the difference between the symptoms that the scales are sampling, HAMD focuses on the somatic and behavioral symptoms, whereas BDI underlines subjective symptoms (24). Longitudinal studies show that the HAMD is a more sensitive measure of symptom change than BDI (25).

In the present research, we sought to answer two questions: To what extent can a model taught with BDI accurately learn the severity of depression? To what extent is it possible to use BDI and HAMD scores together and does using HAMD instead of BDI for some of the training samples improve model performance? Answering the first question is important because the BDI questionnaire is self-reported, so it may have limits in the operation of the model. The answer to the second question is important from a practical point of view. On the one hand, if there are two depression databases, but the severity of depression was measured on a different scale, it is necessary to transform them into a common scale. On the other hand, it is faster to collect a database based on BDI, but at the same time the HAMD scores assess different symptoms of depression, so maybe it would be possible to improve the performance of the acoustic model by adding and using some samples labeled with HAMD scores.

## Materials and methods

### Database

In the present research we used the Hungarian Depressed Speech Database (DEPISDA) (19). The database currently contains speech samples of 218 (144 females and 74 males) Hungarian subjects (depressed and healthy subjects). The collection of speech samples and the selection of subjects are carried out jointly by the Laboratory of Speech Acoustics, Budapest University of Technology and Economics and the Department of Psychiatry and Psychotherapy, Semmelweis University. The speech samples are independent, all patients were recorded only once.

Study inclusion criteria for patients with depression required study participants to be at least 18 years old and BDI score of 14 or higher. Exclusion criteria was ongoing antipsychotic medication use because extrapyramidal symptoms can negatively affect the articulatory system, and conceptualizing and formulating are known to be negatively affected by antipsychotic drugs that block dopamine receptors (26).

Read speech samples were recorded from each subject. Each speaker had to read a tale of about 10 sentences (“The North Wind and the Sun”). BDI score, age, gender, smoking habits, medications taken and other speech-related illnesses and conditions of the subjects were also noted in each case. HAMD score of examined subjects was recorded in 20% of cases, in a total of 43 speakers. Nearly half of the subjects in the database are healthy, while depressed patients cover different degrees of severity of depression almost evenly. In order to achieve our research goals, we divided the database into two sets. *Set I* included samples where only BDI scores were available. *Set II* included samples where both BDI and HAMD scores were available.

To compare HAMD scores and BDI scores, the endpoints of the Hamilton scale categories (minimum 0–7, mild 8–13, moderate 14–18, severe 19–48) were fitted to the endpoints of BDI categories (minimum 0–13, mild 14 –19, moderate 20–28, severe 29–63), and then linear scaling was used within each category, since the relationship is presumably linear within each scale.

**FIGURE 1:**
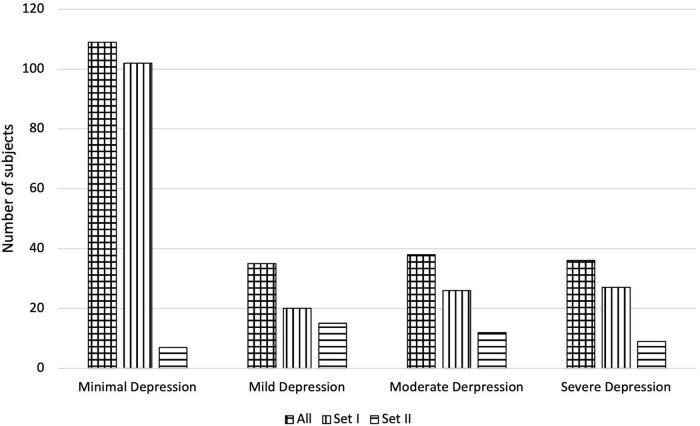
Number of subjects of the applied Depressed Speech Database by H2B category in the case of All, Set I, and Set II.

The rescaled HAMD scores are hereinafter referred to as H2B. An H2B score was then assigned to each sample, which, if a HAMD score was available, was obtained by scaling it, otherwise the BDI score was assigned. Thus, both BDI and H2B scores are available for each sample in each set during further experiments. The main descriptive statistics of the subjects are shown in Table 1, and the distribution of the subjects by depression is shown in Figure 1.

**TABLE 1:** Main descriptive statistics of subjects of the applied Depressed Speech Database (DEPISDA).

		Count	H2B (score)		BDI (score)		Age (year)	

			Mean	Std. Dev.	Mean	Std. Dev.	Mean	Std. Dev.
Set I	Both	175	13.5	12.8	same as for H2B	same as for H2B	44.3	16.0
	Males	62	13.3	11.8	same as for H2B	same as for H2B	43.2	17.9
	Females	113	13.7	13.4	same as for H2B	same as for H2B	44.9	14.9
Set II	Both	43	20.7	7.9	21.4	7.9	34.3	11.7
	Males	12	18.1	7.7	23.9	5.6	35.7	10.4
	Females	31	21.6	7.8	27.4	8.5	33.8	12.4
All	Both	218	15.0	12.3	16.1	13.1	42.3	15.8
	Males	74	14.1	11.4	15.0	11.7	42.0	17.1
	Females	144	15.4	12.8	16.7	13.7	42.4	15.1

### Preprocessing

Speech samples were then normalized to peak amplitude to eliminate any different recording gains. Annotation of speech samples was created using a transcription based force alignment automatic segmentation method (27) valid for several languages. Segmentation and annotation of speech and silence parts were performed, as well as phoneme-level segmentation of speech parts using the SAMPA alphabet. Phoneme-level segmentation is important because calculating certain characteristics at phoneme level improves the accuracy of automatic recognition of depression (9, 28).

The following low-level descriptors (LLDs) were calculated: intensity, fundamental frequency, jitter, shimmer, first and second formant frequencies and their bandwidths and 13 mfcc coefficients. Low-level features were calculated with a 30 ms Hamming window length and with 10 ms timestep. The fundamental frequency, jitter, shimmer, and formant frequencies were calculated from the voiced parts of the speech sample, while the other LLDs were calculated over the entire speech sample. These LLDs were calculated because previous studies have shown that these speech characteristics alter as a result of depression (10, 15).

### Feature extraction

From the LLDs, the actual descriptive features were calculated using the mean, standard deviation and percentile ranges (range formed after leaving the lower and upper 1, 5, 15, and 25% of the ordered values). Some descriptive characteristics were calculated based on more place-dependent methods, from vowel E (Sampa notation for Hungarian “e”) and vowel O (Sampa notation for Hungarian “a”), from all vowels, and over the whole speech part (Table 2), so a total of 282 features were calculated from LLDs.

**TABLE 2:** The used descriptive features calculated from LLDs.

	Calculated from	Total feature number
Fundamental frequency	Voiced parts	6
Intensity	Vowels, whole speech	12
Jitter	E, O, vowels	18
Shimmer	E, O, vowels	18
First and second formant frequencies and their bandwidths	E, O, vowels	72
13 MFCC	Vowels, whole speech	156
Articulation rate	Whole speech	1
Pause ratio	Whole sample	1
Ratio of transients	Whole speech, whole sample	2
Total:		286

Additional features were also calculated, such as articulation rate, pause ratio and the ratio of transient parts on speech parts and on total speech sample. Thus, a total of 286 features were calculated from each speech sample (Table 2).

### Model training and optimization

Support Vector Regression (SVR) (29) was selected as the machine learning method, which is a version of the Support Vector Machines (SVM) family for solving a regression problem (30, 31). We selected this because several studies have shown that using SVMs can give good results in the automatic recognition of depression (8–10). However, their use is not necessary, similar results can be obtained with other machine learning methods (18, 19, 32, 33). The LibSVM (34) SVR implementation was used with radial basis function (rbf) kernel.

The models were trained based on the whole database, as the sample number of *Set II* would not have been sufficient for training purposes. Separate models were trained based on the gender of the subjects, because it improves the automatic recognition of depression (9, 28). Due to the small size of the database, the training and testing of SVR models were implemented with leave-one-out cross-validation (LOOCV), so each sample was selected once as a test set and the model were trained and optimized with the remaining samples. The accuracy of the SVR depends largely on the appropriate choice of the input feature vector and hyperparameters (cost and gamma). Thus, the training and optimization of the models were also implemented using the LOOCV. This double cross-validation is called as nested loop (10, 35), in our case nested LOOCV. The advantage of the method is that the sets of train, development and test are completely independent of each other, thus minimizing the chance of overfitting and so gives a realistic overview of the accuracy and generalizing ability of the given method. In addition, we also give the results with fivefold nested loops at the beginning to compare the nested LOOCV with the classic 80/20 train/test separation as well.

A feature selection process was applied to reduce the feature set size by selecting the 20 features correlating the most with depression severity (highest Pearson correlation) based on the train samples and then applied a fast forward selection (selecting max 20 features) method (9) to reduce redundancy and find the best performing feature set. Finally, the optimization of the hyperparameters (cost and gamma) was realized by grid search method, where the powers of 2 were tried out between −7 and +7.

### Evaluation

Beside Root Mean Square Error (RMSE) as primary evaluation metric, mean absolute error (MAE), Spearman and Pearson correlation coefficients between the original and predicted target scores were also calculated.

Based on the predicted values of the regression method, classification experiments were also performed, where the classification accuracy, sensitivity and specificity metrics were examined. In addition, the receiver operating characteristic (ROC) curve and its area under the curve (AUC) score of the classification models are given.

It is important to note that due to the application of the nested loop, test samples were completely independent of the training and optimization of the models, so the results obtained reflect the real generalizing ability of our method.

## Results

### Depression severity estimation based on Beck Depression Inventory

In the first experimental setup, we performed model training and optimization based on the BDI scores. The best results were obtained with five features in the feature vectors for both males and females using nested LOOCV (RMSE: 11.5 in case of females and 8.0 in case of males). In contrast, for fivefold nested loops, the best results were slightly worse (RMSE: 12.0 in case of females, 8.3 in case of males). Of course, this is not surprising, as in this case the size of the training set is smaller and the model optimization is also more easily overfit, resulting in worse scores during testing. For this reason, only the nested LOOCV evaluation was used in the following.

**FIGURE 2:**
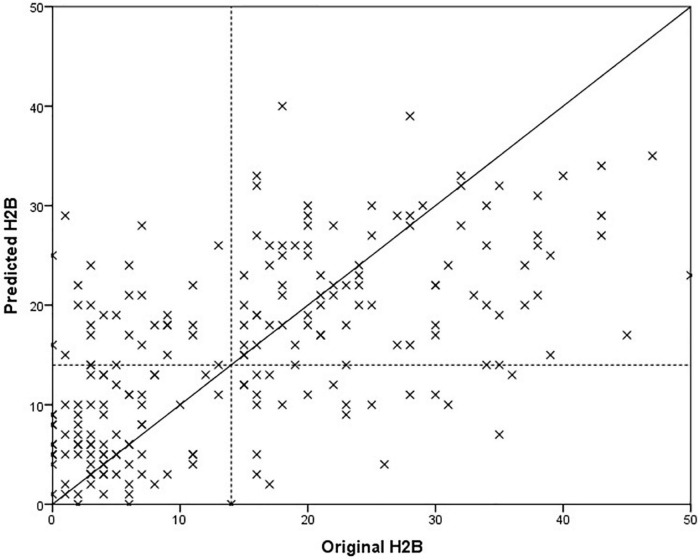
Comparison of predicted and original depression severity scores, when the BDI score was used for training.

Since the model optimization and testing was implemented with a nested loop, it is conceivable that a different model was selected for each test sample. For this reason, we provide those features that were selected in at least 90% of the all cases, however, some of the features are highly correlated with each other, so they are easily interchangeable. For female models, these features were ratio of transients on the whole speech parts, the standard deviation of mfcc6 on vowels, and the mean of mfcc1 over the entire speech parts. For male models, these features were ratio of transients on the whole speech parts and the mean of mfcc4 on the vowels. The models were tested for both BDI and H2B target variables, results are shown in Table 3. Results for male and female sets are marked with “m” and “f,” respectively. Scatter plot of the predicted and the original H2B scores is shown in Figure 2, where the boundary of depression is indicated by a dashed line (H2B = 14), and the line *x* = *y* (perfect decision) was also plotted.

**TABLE 3:** Accuracy of depression prediction, when the BDI scores were used for training.

	Target	RMSE	MAE	Pearson	Spearman
Set I	BDI (=H2B)	10.1 (m: 7.7; f: 11.2)	7.7 (m: 6.2; f: 8.5)	0.63 (m: 0.76; f: 0.56)	0.59 (m: 0.73; f: 0.52)
Set II	BDI	11.6 (m: 9.5; f: 12.4)	8.7 (m: 7.5; f: 9.2)	0.19 (m: 0.32; f: 0.14)	0.24 (m: 0.11; f: 0.15)
	H2B	9.3 (m: 6.0; f: 10.3)	7.1 (m: 4.7; f: 8.0)	0.21 (m: 0.56; f: 0.15)	0.27 (m: 0.55; f:0.25)
All	BDI	10.4 (m: 8.0; f: 11.5)	7.9 (m: 6.4; f: 8.7)	0.61 (m: 0.73; f: 0.56)	0.61 (m: 0.71; f: 0.56)
	H2B	9.9 (m: 7.4; f: 11.0)	7.6 (m: 5.9; f: 8.4)	0.61 (m: 0.76; f: 0.54)	0.61 (m: 0.74; f: 0.54)

Paired samples *T*-test was used to examine whether the difference in mean absolute error between testing on BDI and H2B was significant in case of *Set II*. We got *p* = 0.129, based on which it is not possible to speak of a significant difference. In case of males, it can be observed that the model was able to estimate the severity of depression with a smaller error (*p* = 0.007, using independent samples *T*-test). One possible explanation is that the mean and the standard deviation of the original BDI scores seems smaller for males (males: 15.0 ± 11.7; females 16.7 ± 13.7), and the accuracy of the model was worse for a more severe depression, although no significant difference can be detected in the values of the original BDI scores with an independent sample *t*-test (*p* = 0.381). At first glance, the results obtained on *Set I* and *Set II* testing on BDI do not appear to differ significantly in the case of the RMSE error value (*Set I*: 10.7; *Set II*: 11.6, *p* = 0.370 using independent samples *T*-test); however, a significant deterioration can be observed for the correlation values for *Set II* (*p* < 0.001 using Fisher’s r to z transformation), from this it can be concluded that the performance of the model is different for *Set I* and *Set II*. It is important to note that while *Set I* contained samples from completely healthy to severely depressed, *Set II* contains mainly speech patterns of depressed subjects (Figure 1). From these it can be concluded that the method is primarily able to properly separate the whole scale, but in the case of a narrower range it differentiates less reliably.

**FIGURE 3:**
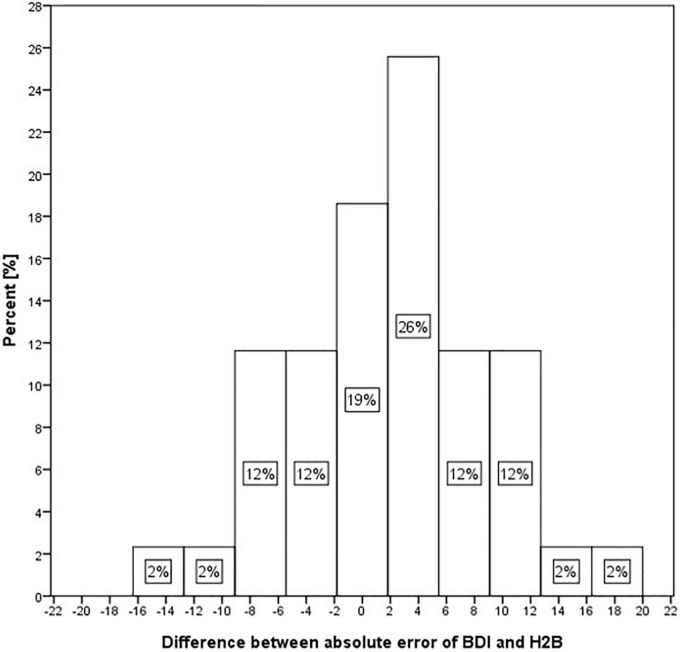
Histogram of differences of absolute error in the case of BDI and H2B for Set II.

Although we could not detect a significant difference in the case of *Set II* (where both BDI and HAMD scores were available), when the predicted values were compared to the BDI or H2B. However, it is possible that this was due to the small number of samples (43), which is why we performed further descriptive analyses. In the case of the MAE error value, we obtained an error value of 8.7 on BDI outcomes opposed to 7.1 for H2B prediction (18% relative improvement). Generally, this means an estimate closer to the H2B score by 1.7 on average. To investigate this in more depth, we looked at the difference in absolute errors for each sample when using the BDI or H2B target variable. The histogram of the differences is shown in Figure 3. Negative difference means a closer result to the BDI, while the positive difference means a closer result to the H2B.

Figure 3 shows that in 19% of the cases, the predicted score shows nearly identical differences from the BDI and H2B scores. However, in 26% of cases it approached H2B with a value of 2–6, in 24% with a value of 6–13, while in a further 4% with a higher value, in contrast only in 12% of cases it approached the BDI with a value of 2–6, in 14% with a value of 6–13, while in a further 2% with a higher value. Based on these, it can be stated that the estimated scores tend to be closer to the H2B score.

### Depression severity estimation based on H2B

**FIGURE 4:**
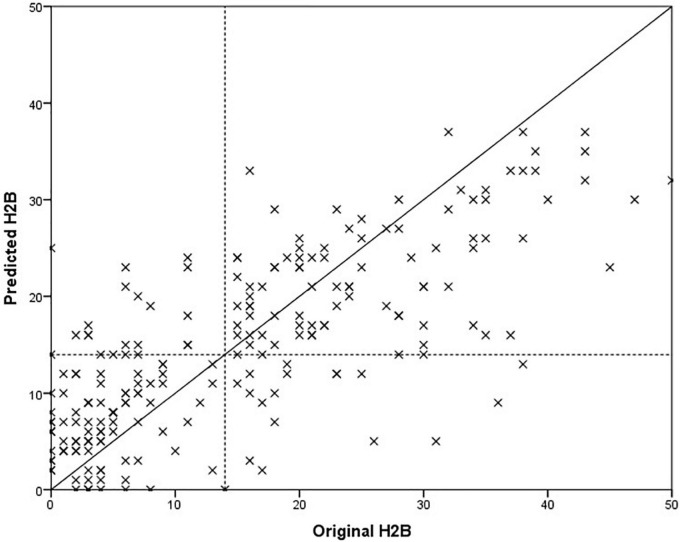
Comparison of predicted and original depression severity scores, when the H2B scores were used for training.

In the previous chapter, it was shown that models trained with BDI tended to estimate H2B more accurately than BDI itself. For this reason, we examined how the accuracy of the method changes if we replace the BDI with H2B for *Set II* (the HAMD was available only for this set, resulting different BDI and H2B scores). Best results were obtained with feature vector of size 7 for males and size 9 for females. As in the previous section, we present the features that were selected in at least 90% of the cases. For female models, these features were both of ratio of transients, standard deviation of intensity on the whole speech parts, mean of mfcc1 on the vowels and mfcc2 on the whole speech parts, standard deviation of mfcc6 on the vowels. For male models, these features were ratio of transients on the whole speech parts, mean of mfcc1 on the whole speech parts and mfcc4 on the vowels. The results are shown in Table 4. A comparison of the predicted scores and the original H2B scores is shown in the Figure 4, where the boundary of depression is indicated by a dashed line (H2B = 14), and the line *x* = *y* (perfect decision) was also plotted.

**TABLE 4:** The accuracy of the depression prediction, when the H2B scores were used for training.

	Target	RMSE	MAE	Pearson Coef.	Spearman Coef.
Set I	H2B	8.2 (m: 5.7; f: 9.3)	6.3 (m: 4.4; f: 7.4)	0.77 (m: 0.88; f: 0.72)	0.72 (m: 0.85; f: 0.65)
Set II	H2B	8.2 (m:3.8; f: 9.4)	6.1 (m: 3.1; f: 7.3)	0.42 (m: 0.87; f: 0.25)	0.46 (m: 0.79; f: 0.32)
All	H2B	8.2 (m: 5.4; f: 9.3)	6.3 (m: 4.2; f: 7.4)	0.75 (m: 0.88; f: 0.69)	0.73 (m: 0.87; f: 0.67)

In the case of set *All*, we obtained an error value of 8.2 RMSE, which is a 17% relative improvement compared to training and optimizing the models purely with BDI (9.9 RMSE). Paired samples *T*-test was used to examine whether this difference was significant or not. We got *p* < 0.001, which means that the significant difference can be claimed at a significance level of 99.9%. There was a similar improvement for *Set I* (19%, *p* < 0.001) and *Set II* (12%, *p* < 0.001). It is important to note that the results improved not only for *Set II* but also for *Set I*, which were still trained based on BDI (for *Set I*, BDI = H2B). Based on this, it can be stated that the automatic model created based on the speech signal is better able to learn H2B than BDI.

The degree of improvement can also be observed by comparing Figures 3, 4, as in the second case the estimated scores are closer to the *x* = *y* line (perfect prediction), and fewer samples were placed in the upper left and lower right rectangles, which represent erroneous recognitions (upper left: false positive, lower right: false negative).

### Comparing the precision of acoustic model and Beck Depression Inventory questionnaire

Since both the BDI and HAMD scores were available for *Set II*, this set allowed us to compare the accuracy of the BDI questionnaire and the developed acoustic model. Since the set includes mainly depressed patients, only limited conclusions can be drawn from this comparison, and it is not a good descriptor of the accuracy of the BDI questionnaire in general. However, it may be interesting to compare the two methods, as the long-term goal is to develop a diagnostic support tool that may even surpass the usability of the BDI questionnaire.

**FIGURE 5:**
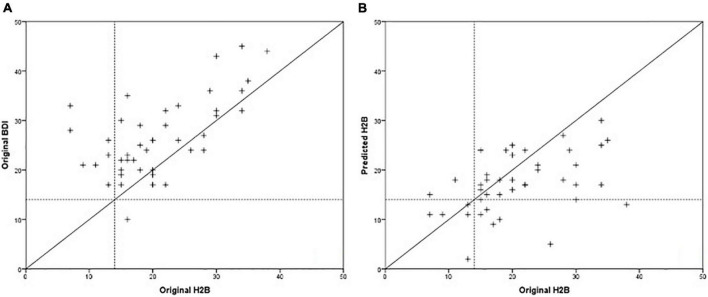
Comparison of BDI scores **(A)** and predicted scores of the acoustic model **(B)** with the H2B scores.

Based on Table 5 and Figure 5, it is not possible to clearly determine which method is more suitable for predicting the severity of depression. While the acoustic model has slightly lower RMSE and MAE error values (*p* = 0.513, paired samples *T*-test), the BDI questionnaire has a better correlation with the H2B (converted HAMD) score (*p* = 0.094, using Fisher’s r to z transformation), so it has a better ability to differentiate in this range of depression severity. Comparing the mean errors, it can be said that while the BDI questionnaire overestimates the severity of depression, the acoustic model underestimates it.

**TABLE 5:** Accuracy of the acoustic model and BDI questionnaire.

	RMSE	MAE	Mean Error	Pearson Coef.	Spearman Coef.
BDI Questionnaire	8.8	6.9	5.7	0.63	0.55
Acoustic model	8.2	6.1	-3.3	0.42	0.46

It can also be observed that in the case of the acoustic model, the majority of significant errors occur in the case of severe depression (H2B > 28), and the maximum predicted score was around 30. This may be due to the insufficient number of severe depressed patients in the database. However, in the 7 cases where subjects received moderate to severe depression scores based on the BDI questionnaire, the speech-based method correctly predicted them to be as non-depressed or mildly depressed.

### Distinguishment between depressed and healthy subjects

**FIGURE 6:**
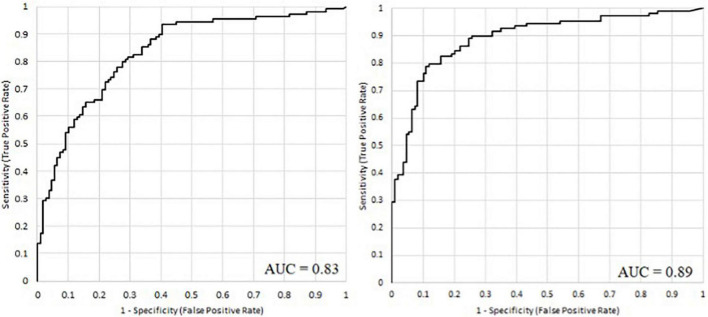
The ROC curve of the acoustic models when models were trained with BDI **(left)** and with H2B scores **(right)**.

It has been shown that it is possible to accurately predict the severity of depression based on speech signal processing. However, from the magnitude of the RMSE and MAE error values, it is difficult to infer the applicability of the method directly, and since the long-term goal is to support the diagnosis of depression, we examined how accurately the model can distinguish between depressed and healthy subjects. With the help of the regression method, classification can also be implemented, since a comparator value can be given, above which the given person is classified as depressed, while below it he is classified as non-depressed. Based on this, we examined how accurately it is possible to distinguish between depressed and non-depressed subjects using models trained with BDI and H2B. Figure 6 shows the ROC curves and the AUC values of the two models, and Table 6 shows their classification sensitivity, specificity as well as at their maximum classification accuracy, and when the comparator limit is set to achieve sensitivity value of at least 90%.

**TABLE 6:** The accuracy of the classification of depressed and healthy subjects, when H2B or BDI scores were used for training.

	Training variable	Classification accuracy	Sensitivity	Specificity
At maximum classification accuracy	BDI	76%	80%	72%
	H2B	84%	79%	89%
At 90% sensitivity	BDI	75%	90%	62%
	H2B	81%	90%	73%

Using the Wilcoxon signed ranks test, we examined whether the difference is significant in the case of maximum accuracy, and we obtained a value of *p* = 0.022, which means that the difference is significant. Based on the examination, it can be stated that the use of H2B in training also improves the classification, as we achieved higher AUC (area under curve) value (0.89), higher maximum classification accuracy (84%), higher specificity (73%) and classification accuracy (81%) at 90% sensitivity.

## Discussion

As the prevalence of depression is increasing worldwide, burdening the health care systems and the global economy, screening systems for early diagnostics are much more needed. Analyzing speech samples can be an easy, cheap and widely available tool in screening process of depression, throughout general medical practices (10). In our study we try to achieve a more accurate, more sensitive model to determine depression by speech samples.

We primarily sought to answer how a limited target variable can be used to estimate the actual severity of depression or to differentiate between depressed and non-depressed subjects. BDI score may be a good descriptor of the severity of depression; however, since it can be determined using a self-reported questionnaire, due to exaggeration and concealment of symptoms it cannot be considered as a fully objective descriptor of the severity of depression. However, determining the BDI score is faster and less resource intensive, making it easier to create a large speech database based, which is a prerequisite for accurate machine learning models.

Hamilton Rating Scale for Depression score, as a clinician-administered scale, assess externally determinable symptoms of depression (like somatic and behavior symptoms), and a more sensitive measure of symptom change, as it is determined by a psychiatrist, also depends on the clinician’s training and subjective judgment.

During the research, we had access to a large speech database containing speech samples from depressed and healthy subjects. The database contains the BDI score of each subject and the HAMD score of 20% of the subjects. This fact provided us with an opportunity to compare the usability of the BDI and the HAMD for the training of the models of the automatic recognition of depression based on speech signal processing. For comparison, HAMD scores were converted to the BDI scale by fitting the endpoints of the two scale categories, and linear scaling was performed within each category to obtain the converted HAMD score: H2B.

We have shown that when models were trained with BDI scores, the predicted scores are on average 1.7 points closer to the converted HAMD scores than to the BDI scores, despite the fact that the BDI scores themselves approximate the HAMD scores quite accurately (MAE 6.9). Of course, while interpreting the results we had to take it into account that we had speech sample from 43 subjects available to perform the experiment, of which 36 subjects were depressed. Thus, the study may be worth performing on a larger set of samples in the future that includes a balanced number of depressed and healthy subjects.

We have shown that changing the BDI scores to HAMD scores at the training improves the automatic prediction and recognition of depression. We examined this by using the converted HAMD scores (H2B) instead of the BDI scores of the 43 samples available to us. Thus, for regression, the RMSE error decreased from 9.9 to 8.2, which is a 17% relative improvement, while the MAE error decreased from 7.6 to 6.3, which is a 17% relative improvement also, which is significant at 99.9% significance level. In the case of classification, accuracy increased from 81 to 84%, which is a 10% relative improvement in the number of erroneously decided subjects.

We also examined whether the acoustic model, or the BDI scale, provides a more accurate prediction of HAMD scores. In the experiment, we found that while the acoustic model estimates HAMD scores (i.e., severity of depression) with smaller RMSE and MAE values than the BDI scale, BDI scores show a higher correlation with HAMD scores than the predicted ones. Thus, it is not possible to clearly determine which is more suitable for a more accurate estimate of the severity of depression; however, it can be concluded that the usability of the acoustic model is comparable to the BDI scale. It is worth noting that data augmentation would likely to further slightly improve the results, for example, if the recordings are splitted into smaller segments (36) and then nested speaker LOOCV evaluation is applied.

The results of our study show that screening by acoustic biomarkers, the diagnosis of depression can be recognized earlier, and with a machine learning based automatic decision system, it can be used widely as a diagnostic tool in the general medical practices. A machine learning based system using acoustic biomarkers of depression can have public applicability, with the possibility of screening the general population for depression by easily accessible, cheap methods, e.g., smartphone applications, webpages. The early recognition of depression by speech analysis can help to refer the patients to psychiatrists earlier to start the proper treatment. The early recognition of depression can improve the quality of life, decrease the mortality and the average length of stay in hospitals, in this way can reduce the economic cost and ease the health care systems. Another application of speech analysis in the diagnosis of depression is to follow the patient’s recovery under treatment and to compare effectivity of variable therapies.

In the near future, we would like to test the method we have developed in practice in primary healthcare, as well as further expand the database to create even more accurate models and better compare the usability of the acoustic model with the BDI scale.

## Data availability statement

The raw data supporting the conclusions of this article will be made available by the authors, without undue reservation.

## Ethics statement

The studies involving human participants were reviewed and approved by the Semmelweis University Regional and Institutional Committee of Science and Research Ethics. The patients/participants provided their written informed consent to participate in this study.

## Author contributions

BH-D, GK, DS, KV, and LS contributed to the design and implementation of the research. BH-D, GK, and DS collected data on the patients. GK performed the computations and statistical analysis. BH-D and GK wrote the manuscript. DS, KV, and LS supervised the project. All authors provided critical comments and approved the final version of the manuscript.

## References

[1] World Health Organization [WHO]. *Depression fact sheet Geneva, Switzerland.* Geneva: WHO (2020).

[2] World Health Organization [WHO]. *The global burden of disease: 2004 update.* Geneva: WHO (2008).

[3] Stuart H. Reducing the stigma of mental illness. *Glob Ment Health (Camb).* (2016) 3:e17. 10.1017/gmh.2016.11 28596886 PMC5314742

[4] Lin CH Lu MJ Wong J Chen CC. Comparison of physician-rating and self-rating scales for patients with major depressive disorder. *J Clin Psychopharmacol.* (2014) 34:716–21. 10.1097/JCP.0000000000000229 25310200

[5] Lakhan SE Vieira K Hamlat E. Biomarkers in psychiatry: Drawbacks and potential for misuse. *Int Arch Med.* (2010) 3:1. 10.1186/1755-7682-3-1 20150988 PMC2820448

[6] Schmidt HD Shelton RC Duman RS. Functional biomarkers of depression: Diagnosis, treatment, and pathophysiology. *Neuropsychopharmacology.* (2011) 36:2375–94. 10.1038/npp.2011.151 21814182 PMC3194084

[7] de Aguiar Neto FS Rosa JLG. Depression biomarkers using non-invasive EEG: A review. *Neurosci Biobehav Rev.* (2019) 105:83–93. 10.1016/j.neubiorev.2019.07.021 31400570

[8] Cummins N Scherer S Krajewski J Schnieder S Epps J Quatieri TF. A review of depression and suicide risk assessment using speech analysis. *Speech Commun.* (2015) 71:10–49. 10.1016/j.specom.2015.03.004

[9] Kiss G Vicsi K. Mono-and multi-lingual depression prediction based on speech processing. *Int J Speech Technol.* (2017) 20:919–35. 10.1007/s10772-017-9455-8

[10] Low DM Bentley KH Ghosh SS. Automated assessment of psychiatric disorders using speech: A systematic review. *Laryngoscope Investigat Otolaryngol.* (2020) 5:96–116. 10.1002/lio2.354 32128436 PMC7042657

[11] Low L-SA Maddage NC Lech M Sheeber LB Allen NB. Detection of clinical depression in adolescents’ speech during family interactions. *IEEE Trans Biomed Eng.* (2010) 58:574–86. 10.1109/TBME.2010.2091640 21075715 PMC3652557

[12] Alghowinem S Goecke R Wagner M Epps J Breakspear M Parker G editors. From joyous to clinically depressed: Mood detection using spontaneous speech. *Proceedings of the twenty-fifth International Florida artificial intelligence research society conference. Association for the Advancement of Artificial Intelligence (AAAI) Citeseer.* Menlo Park, CA: AAAI (2012). p. 141–6.

[13] Pampouchidou A Simantiraki O Vazakopoulou C-M Chatzaki C Pediaditis M Maridaki A editors. Facial geometry and speech analysis for depression detection. *Proceeding of the 2017 39th annual international conference of the IEEE engineering in medicine and biology society (EMBC).* Piscataway, NJ: IEEE (2017). 10.1109/EMBC.2017.8037103 29060147

[14] Huang Z Epps J Joachim D Stasak B Williamson JR Quatieri TF. Domain adaptation for enhancing Speech-based depression detection in natural environmental conditions using dilated CNNs. *Interspeech.* (2020) 2020:4561–5. 10.21437/Interspeech.2020-3135

[15] Kiss G Vicsi K editors. Comparison of read and spontaneous speech in case of automatic detection of depression. *Proceeding of the 2017 8th IEEE international conference on cognitive infocommunications (CogInfoCom).* Piscataway, NJ: IEEE (2017). 10.1109/CogInfoCom.2017.8268245

[16] Cummins N Sethu V Epps J Williamson JR Quatieri TF Krajewski J. Generalized two-stage rank regression framework for depression score prediction from speech. *IEEE Trans Affect Comput.* (2017) 11:272–83. 10.1109/TAFFC.2017.2766145

[17] Rejaibi E Komaty A Meriaudeau F Agrebi S Othmani A. Mfcc-based recurrent neural network for automatic clinical depression recognition and assessment from speech. *arXiv* *[Preprint]*. (2019):190907208. 10.48550/arXiv.1909.07208 35895330

[18] Williamson JR Young D Nierenberg AA Niemi J Helfer BS Quatieri TF. Tracking depression severity from audio and video based on speech articulatory coordination. *Comput Speech Lang.* (2019) 55:40–56. 10.1016/j.csl.2018.08.004

[19] Kiss G Jenei AZ editors. Investigation of the accuracy of depression prediction based on speech processing. *Proceeding of the 2020 43rd international conference on telecommunications and signal processing (TSP).* Piscataway, NJ: IEEE (2020). 10.1016/j.cmpb.2021.106433

[20] Lopez-Otero P Docio-Fernandez L. Analysis of gender and identity issues in depression detection on de-identified speech. *Comput Speech Lang.* (2021) 65:101118. 10.1016/j.csl.2020.101118

[21] Carmody TJ Rush AJ Bernstein I Warden D Brannan S Burnham D The montgomery asberg and the Hamilton ratings of depression: A comparison of measures. *Eur Neuropsychopharmacol.* (2006) 16:601–11. 10.1016/j.euroneuro.2006.04.008 16769204 PMC2151980

[22] Richter P Werner J Heerlein A Kraus A Sauer H. On the validity of the Beck depression inventory. A review. *Psychopathology.* (1998) 31:160–8. 10.1159/000066239 9636945

[23] Bukumiric Z Starcevic V Stanisavljevic D Marinkovic J Milic N Djukic-Dejanovic S Meta-analysis of the changes in correlations between depression instruments used in longitudinal studies. *J Affect Disord.* (2016) 190:733–43. 10.1016/j.jad.2015.10.054 26606717

[24] Lambert MJ Hatch DR Kingston MD Edwards BC. Zung, Beck, and Hamilton Rating Scales as measures of treatment outcome: A meta-analytic comparison. *J Consult Clin Psychol.* (1986) 54:54–9. 10.1037//0022-006x.54.1.54 3958302

[25] Edwards BC Lambert MJ Moran PW McCully T Smith KC Ellingson AG. A meta-analytic comparison of the Beck Depression Inventory and the Hamilton Rating Scale for Depression as measures of treatment outcome. *Br J Clin Psychol.* (1984) 23(Pt 2):93–9. 10.1111/j.2044-8260.1984.tb00632.x 6722384

[26] de Boer JN Voppel AE Brederoo SG Wijnen FNK Sommer IEC. Language disturbances in schizophrenia: The relation with antipsychotic medication. *NPJ Schizophr.* (2020) 6:24. 10.1038/s41537-020-00114-3 32895389 PMC7477551

[27] Kiss G Sztahó D Vicsi K editors. Language independent automatic speech segmentation into phoneme-like units on the base of acoustic distinctive features. *Proceeding of the 2013 IEEE 4th international conference on cognitive infocommunications (CogInfoCom).* Piscataway, NJ: IEEE (2013). 10.1109/CogInfoCom.2013.6719169

[28] Cummins N Vlasenko B Sagha H Schuller B editors. Enhancing speech-based depression detection through gender dependent vowel-level formant features. *Proceeding of the Conference on artificial intelligence in medicine in Europe.* Berlin: Springer (2017). 10.1007/978-3-319-59758-4_23

[29] Drucker H Burges CJ Kaufman L Smola A Vapnik V. Support vector regression machines. *Adv Neural Inform Process Syst.* (1997) 9:155–61.

[30] Cortes C Vapnik V. Support vector machine. *Machine Learning.* (1995) 20:273–97. 10.1007/BF00994018

[31] Suykens JA Vandewalle J. Least squares support vector machine classifiers. *Neural Process Lett.* (1999) 9:293–300.

[32] Kiss G Tulics M Sztaho D Esposito A Vicsi K FaundezZanuy M Language independent detection possibilities of depression by speech. *Recent Adv Nonlinear Speech Process.* (2016) 48:103–14. 10.1007/978-3-319-28109-4_11

[33] He L Cao C. Automated depression analysis using convolutional neural networks from speech. *J Biomed Inform.* (2018) 83:103–11. 10.1016/j.jbi.2018.05.007 29852317

[34] Chang C-C Lin C-J. LIBSVM: A library for support vector machines. *ACM Trans Intell Syst Technol (TIST).* (2011) 2:1–27. 10.1145/1961189.1961199

[35] Gosztolya G Vincze V Tóth L Pákáski M Kálmán J Hoffmann I. Identifying mild cognitive impairment and mild Alzheimer’s disease based on spontaneous speech using ASR and linguistic features. *Comput Speech Lang.* (2019) 53:181–97. 10.1016/j.csl.2018.07.007

[36] Rozmán P Sztahó D Kiss G Jenei AZ. Automatic recognition of depression and Parkinson’s disease by LSTM networks using sample chunking. *Proceeding of the 2021 IEEE 12th international conference on cognitive infocommunications (CogInfoCom).* Piscataway, NJ: IEEE (2021).

